# Percent depth‐dose distribution discrepancies from very small volume ion chambers

**DOI:** 10.1120/jacmp.v16i2.5230

**Published:** 2015-03-08

**Authors:** Vikren Sarkar, Brian Wang, Hui Zhao, Bart Lynch, Joshua A. James, Kiernan T. McCullough, Bill J. Salter

**Affiliations:** ^1^ Department of Radiation Oncology University of Utah Salt Lake City UT; ^2^ Department of Radiation Oncology University of Louisville Louisville KY; ^3^ Center for Cancer Care Huntsville AL; ^4^ Colorado Associates in Medical Physics Colorado Springs CO USA

**Keywords:** microionization chambers, beam scanning, electrometer bias, percent depth dose

## Abstract

As very small ion chambers become commercially available, medical physicists may be inclined to use them during the linear accelerator commissioning process to better characterize the beam in steep dose gradient areas. For this work, a total of eight different ion chambers (volumes from 0.007 cc to 0.6 cc) and four different scanning systems were used to scan PDDs at both +300V and −300V biases. We observed a reproducible, significant difference (overresponse with depth) in PDDs acquired when using very small ion chambers, with specific bias/water tank combinations — up to 5% at a depth of 25 cm in water. This difference was not observed when the PDDs were sampled using the ion chamber in static positions in conjunction with an external electrometer. This suggests noise/signal interference produced by the controller box and cable system assemblies, which can become relatively significant for the very small current signals collected by very small ion chambers, especially at depth as the signal level is even further reduced. Based on the results observed here, the use of very small active volume chambers under specific scanning conditions may lead to collection of erroneous data, introducing systematic errors into the treatment planning system. In case the use of such a chamber is required, we recommend determining whether such erroneous effect exists by comparing the scans with those obtained with a larger chamber.

PACS numbers: 87.56.bd, 87.56.Fc, 87.56.Da

## I. INTRODUCTION

The commissioning of typical treatment planning systems requires measurement of a variety of cross profiles and percent depth‐dose (PDD) curves for multiple field sizes, typically using an ion chamber in a water tank. Due to the finite size of the typical ion chamber, accurate characterization of beam edges can be challenging, with variable extents of broadening of the beam's edges occurring as a function of the dimensions of the active volume.^1^, ^2^ This is especially problematic when scanning beams of small size where the beam profile is rapidly changing. Due to their small active area dimensions, diodes have been recommended for such scans^3^, ^4^, ^5^ but diodes can exhibit overresponse at lower energies, a condition that is more prevalent at depth due to beam softening.^6^, ^7^, ^8^ In addition, diodes also suffer from angular, temperature, and dose‐rate dependence and, thus, the literature has conflicting recommendations on the use of diodes for beam data acquisition.^9^, ^10^

Diamond detectors do not exhibit many of these dependence issues of diode detectors and appear to be well suited for small field beam data measurements.^11^, ^12^, ^13^ Unfortunately, diamond detectors are more expensive than other detectors and can be prohibited from common clinical usage, especially in smaller centers. Recently, plastic scintillation detectors (PSD) have emerged to be a strong candidate to characterize radiation beams for small field dosimetry.^14^, ^15^, ^16^ However, the relative novelty of PSDs means that their suitability for use in beam scanning is still being investigated.

Lately, several so‐called ‘micro’ ion chambers have become commercially available.^(17)^ Because these chambers have relatively small physical active volumes, they can significantly reduce the degree of distortion in beam edge representation that results from the previously mentioned finite size detector effect.^4^, ^18^ Additionally, since ion chambers do not typically exhibit energy dependence, they will not lead to misrepresentation of depth‐dose curves, as they encounter a larger proportion of low energy scatter at depth. It is therefore not surprising that medical physicists are considering use of these newer, microionization chambers in their efforts to accurately commission their treatment planning systems.

The American Association of Physicists in Medicine's (AAPM) Task Group 106 report^(5)^ provides guidelines on the process of commissioning a linear accelerator. The report explains that percent depth‐dose (PDD) scans acquired may vary depending on the detector used, and also alludes to the fact that PDD measurements can change depending on the bias that is applied to the ion chamber. The purpose of our work is to present, explore, and quantify a nontrivial anomalous behavior that is observed when microchambers are used during specific beam scanning situations to acquire PDD curves. Awareness of this anomaly, and the specific circumstances under which it can occur, should help medical physicists avoid introducing erroneous data into the treatment planning system commissioning process.

## II. MATERIALS AND METHODS

The PDD data presented here were acquired using multiple combinations of ion chambers and scanning water tanks, as well as electrometers/controllers, as described in Table 1. All PDD curves were acquired for a $10\times 10\;{\text{cm}}^{2}$ field size. Given that the signal level obtained when using the very small chambers was relatively low, all scans were obtained at the lowest scan speed allowable (15 mm/s) and data were collected using a 2 mm spacing.

In Setup A, a total of six ion chambers with active volumes from 0.007 to 0.6 cc were used to scan the same beam using both +300V and −300V bias settings provided by a Scanditronix/Wellhöfer (IBA Dosimetry, Schwarzenbruck, Germany) scanning system with the RFA300 MCU controller on a Varian Clinac 21CD (Varian Medical Systems, Palo Alto, CA). The purpose of this experiment was to investigate the role of ion chamber size on the anomaly being investigated.

Based on the results obtained for this experiment, all subsequent experiments used only the A16 microchamber which showed the greatest extent of the anomalous behavior described in this work. The reasoning was that the chamber that showed the most discrepancy would allow us to more easily determine whether any of the following tests eliminated the observed effect.

Setup B used the same scanning system as Setup A, except that three different chambers of the same model of microchamber were used (Exradin A16; 0.007 cc, Standard Imaging, Middleton, WI) to scan a 6 MV beam using both +300V and −300V bias settings. These scans were deemed valuable for characterizing the variations in the measured PDD anomaly as a function of manufacturing variations for the same model chamber. Both experiments of Setup A and B were repeated over at least three separate days to verify that the same effect is observed each time. Each of the repeated experiments confirmed the existence of the anomalous effect.

Setup C used a single Exradin A16 chamber in conjunction with four different scanning systems to obtain PDDs using +300V and −300V bias. In addition to the original Scanditronix/Wellhöfer system with the RFA300 controller, the following three systems were also used: 1) the IBA Blue Phantom^2^ scanning tank (IBA Dosimetry) with the CU500E controller on a Varian TrueBeam linear accelerator; 2) the PTW MP3‐M Therapy Beam Analyzer (PTW, Freiburg, Germany) scanning tank with PTW Tandem T10015 controller on a Varian TrueBeam linear accelerator; and 3) the Standard Imaging DoseView 3D scanning system (Standard Imaging Inc, Middleton, WI) on a Varian Clinac iX. In all cases, a larger chamber was also used to scan the PDD using the two different bias settings. This experiment, using four different model water scanning tanks from three manufacturers, was aimed at exploring the role of different water tank scanning systems in the manifestation of the anomaly.

**Table 1:** Various combinations of ion chambers and scanning tanks used for the $10\times 10\;{\text{cm}}^{2}$ PDD acquisitions

*Setup*	*Water Tank*	*Electrometer / Controller*	*Ion Chamber Used*	*Ion Chamber Volume (cc)*	*Energy* ^a^
A	Scanditronix/Wellhofer RFA‐300	RFA‐300 MCU	Exradin A16 Wellhofer IC04	0.007 0.04	6X
			Exradin A1SL PTW 31010 PTW 31013 PTW N30013	0.053 0.125 0.3 0.6	
B	Scanditronix/Wellhofer RFA‐300	RFA‐300 MCU	Exradin A16 (3 chambers)	0.007	6X
	IBA Blue Phantom^2^	CU500E	Exradin A16 IBA CC13	0.007 0.13	
C	Scanditronix/Wellhofer RFA‐300	RFA‐300 MCU	Exradin A16 PTW 31010	0.007 0.125	6X
	PTW MP3‐M Therapy Beam Analyzer	PTW Tandem T10015	Exradin A16 PTW 31010	0.007 0.125	
	Standard Imaging DoseView 3D	DoseView 3D Electrometer	Exradin A16 Exradin A28	0.007 0.125	
D	Scanditronix/Wellhofer RFA‐300	RFA‐300 MCU	Exradin A16	0.07	6X, 6FFF, 10X, 10 FFF
E	Scanditronix/Wellhofer RFA‐300	RFA‐300 MCU CNMC K602 Standard Imaging Max4000	Exradin A16	0.007	6X

^a^ 6X refers to a 6 MV flattened beam; 6FFF represents a 6 MV flattening filter‐free beam; 10X and 10FFF refer to a 10 MV flattened and flattening filter‐free beam, respectively.

In Setup D, the original Scanditronix/Wellhöfer scanning system with the RFA300 MCU controller, along with the A16 chamber, was used to scan PDDs under both positive and negative bias conditions, using flattened and flattening filter‐free (FFF) beams of two energies (6 and 10 MV). This experiment was aimed at determining whether the beam energy/spectrum had any effect on the observed anomaly. The data for the unflattened beams were obtained using a Varian TrueBeam linear accelerator.

Finally, in Setup E, the A16 chamber was used in conjunction with the original Scanditronix/Wellhöfer system to measure the PDD curve both dynamically (i.e., scanned) and statically (i.e., values acquired when the chamber was stationary). This experiment was useful for isolating any variations in the measured PDD anomaly as a function of a moving versus static chamber. Additionally, to allow for characterization of the component of the anomaly that might be attributable to the water scanner's internal electrometer system, PDDs were obtained statically using two different versions of scanner‐independent electrometers (CNMC Model MK602 (CNMC Company, Nashville, TN) and Standard Imaging Model Max4000).

All comparative measurements were performed sequentially, relative to each other, with no significant time lapse or change in setup other than the chamber, the signal gain used, and the positive/negative bias variation. A reference chamber was used for all scans to eliminate linac output variations.

## III. RESULTS

**Figure 1:**
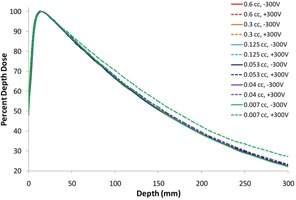
PDDs of $10\times 10\;{\text{cm}}^{2}$, 6 MV beam obtained using six different volume ion chambers (0.007 cc‐0.6 cc) operated with a bias of +300V (dashed line) and −300V (solid line) in the Scanditronix/Wellhöfer water tank with RFA‐300 electrometer controller (i.e., Setup A). Note that the smallest volume chamber (0.007 cc), when scanned with a positive bias, differs nontrivially from PDDs acquired with larger chambers (of either bias), and that the difference increases with depth (4.7% difference at 25 cm depth). Note also that the next smallest chamber (0.04 cc), when also scanned with positive bias, differs slightly from the larger chambers' PDDs, also increasing with depth.

Figure 1 shows the results from scanning the percent depth‐dose (PDD) curve of a $10\times 10\;{\text{cm}}^{2}$, 6 MV beam in the Scanditronix/Wellhöfer water tank with RFA‐300 electrometer controller, at two biases using six different chambers with different active volumes (i.e., Setup A).

As seen in Fig. 1, the PDD curves agree very well with each other, except for those from the two smallest volume chambers, when scanned using the +300V bias. The disagreement for these two chambers can be seen to increase with depth, with greatest variation, 4.7% at 25 cm depth, observed for the smallest chamber (0.007 cc).

**Figure 2:**
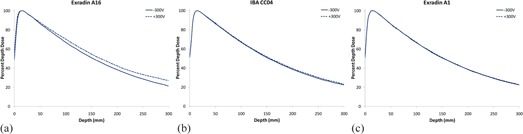
PDDs obtained with the three smallest chambers using the Wellhöfer tank. 2(a): Scanned PDDs obtained using an Exradin A16 chamber (0.007 cc) with both +300V and −300V biases. Note that the +300V bias scan rises above the −300V scan increasingly with depth. 2(b): Scanned PDDs obtained with IC04 chamber (0.04 cc) with both +300V and −300V biases. Note that the +300V bias scan rises above the −300V scan increasingly with depth, although not as dramatically as for the much smaller 0.007 cc chamber. 2(c): Scanned PDDs obtained with A1 chamber (0.053 cc). Note that the +300V bias scan appears to track only very slightly above the −300V scan. All scans were obtained using the Scanditronix/Wellhöfer water tank with the RFA‐300 electrometer.

Figure 2 shows direct comparison of +300V and −300V scans acquired in the Scanditronix/Wellhöfer water tank with RFA300 Electrometer‐Controller, for each of the three smallest chambers studied here (A16−0.007 cc; CC04−0.04 cc;A1−0.053 cc). Figure 2 confirms that, for these three chambers, the negative bias scans (solid lines) agree well with the larger volume chambers, when either bias is used, suggesting the negative bias scans are likely to represent the ‘true’ PDD curve. When using the positive bias, however, the scans (dashed lines) rise steadily above the negative bias scans as a function of depth, with the smallest chamber showing the greatest difference, and the difference from presumed ‘truth’ increasing as chamber size decreases.

**Figure 3:**
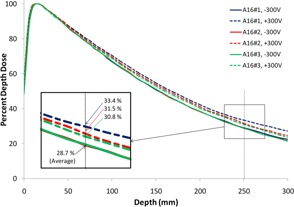
Comparison of 6 MV PDDs obtained using three different versions of the same model of A16 microionization chambers, scanned in the Scanditronix/Wellhöfer water tank with RFA‐300 electrometer controller and operated with biases of both −300V and +300V. Note that all three A16 chambers manifest the ‘reverse‐bias’ anomaly, with differences from negative bias scans ranging from 2.1% to 4.7%, absolute.

Figure 3 shows the results of measuring PDDs using three different A16 model chambers, with both +300V and −300V biases, for the purposes of exploring the potential role of small variations in manufacturing reproducibility within the same model chamber. All three scans obtained at −300V overlay each other within 1% (solid lines), but all scans obtained at +300V (dashed lines) show the anomaly, with nontrivial differences from the negative bias scans.

**Figure 4:**
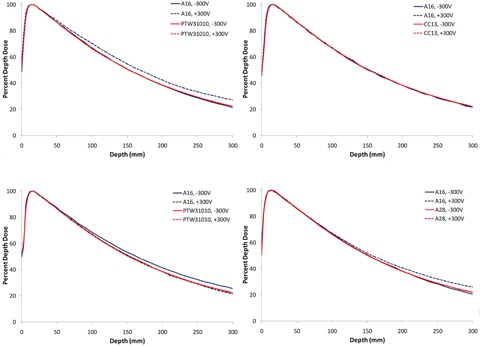
Results of measuring 6 MV PDDs using the same A16 microchamber operated with a bias of ± 300V in three different water tank systems. Also included in the scans are results of PDDs scanned at the two biases for a larger chamber. (a) Scans performed in the Scanditronix/Wellhöfer RFA300 tank; (b) Scans performed in the Blue Phantom^2^ tank; (c) Scans done with the PTW MP3‐M tank; (d) Scans done with the Standard Imaging DoseView 3D with the DoseView 3D electrometer.

Figure 4 shows the results of using an A16 0.007 cc ion chamber (positive 300V and negative 300V) to scan PDDs using four different water scanning systems. Each water scanning tank was used with its respective vendor‐supplied internal electrometer system. The same version A16 chamber (serial #XAA122235) was used for all eight scans. It is apparent in Fig. 4(b) that the reverse‐bias anomaly is not manifested with the A16 chamber when scanned with the Blue Phantom^2^ scanning water tank and internal electrometer, in that the positive and negative bias scans overlay each other identically. Note, alternatively, in 4(a), 4(c), and 4(d), that the reverse‐bias anomaly does manifest for the Scanditronix/Wellhöfer, PTW MP3‐M, and Standard Imaging DoseView 3D water tank systems, in that the positive and negative bias scans show nontrivial differences, appearing largest for the RFA‐300 tank, and increasing as a function of depth for all three systems. Also noteworthy is the fact that, while the anomaly is observed when using the positive bias in the Scanditronix/Wellhöfer tank, the anomaly shows up when the negative bias is used in the PTW tank.

**Figure 5:**
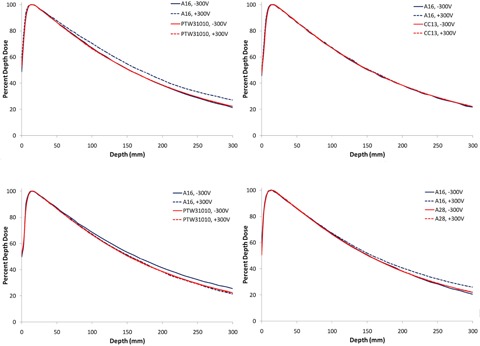
PDDs from flattened and flattening filter‐free beams of both 6 MV and 10 MV energies, obtained using an A16 microchamber, operated with a bias of ± 300V in the Scanditronix/Wellhöfer scanning water tank with RFA‐300 electrometer controller. (a) 6 MV flattened; (b) 6 MV unflattened; (c) 10 MV flattened; (d) 10 MV unflattened.

Figure 5 presents the results of scanning PDD curves for both flattened and unflattened (FFF) beams using the A16 (0.007 cc) chamber. Interestingly, while the reverse‐bias anomaly is observed for both 6 and 10 MV, the magnitude of the anomaly is reduced for the FFF beams.

## IV. DISCUSSION

We observed PDD discrepancies as large as 4.7% at 25 cm depth for the 0.007cc A16 chamber (serial #XAA122235), in combination with a positive scanning bias and the Scanditronix/Wellhöfer system with internal RFA‐300 electrometer controller. To confirm that the discrepancy was not due to a manufacturing defect in the particular version of A16 chamber initially used, we repeated the experiment (Setup B) with two additional versions of the Model A16 chamber, and it is apparent from Fig. 3 that the nontrivial measurement difference is still present, with percent differences of 2.1% (serial # XAA041005), 2.8% (serial #XAA121841), and 4.7% (serial #XAA122235) at 25 cm depth for each of the three A16 chambers used here, thus eliminating the possibility that a chamber manufacturing defect caused the anomaly. The variations in degree of manifestation of the phenomenon for different instances of the same model chamber do, however, suggest the phenomenon is dependent not just on the chamber design but also on subtleties in the performance of each individual chamber.

We then investigated the potential role of the particular scanning system/electrometer used in manifesting the measurement anomaly. It is apparent from Fig. 4 that the scanning tank, with vendor supplied electrometer, does play a role in manifesting the measurement difference, with PDD percentage differences ranging from almost zero (+0.3%) for the Blue Phantom^2^ system, to +3.5% for the PTW MP3‐M system, to +3.9% for the Standard Imaging DoseView 3D system, to +4.7% for the Scanditronix/Wellhöfer with RFA‐300 electrometer controller combination, all at a depth of 25 cm.

Our interpretation of the scanning system's role in manifesting the measurement anomalies was that some form of noise/interference from the scanner's motor/controller/electrometer system was potentially being registered/collected during measurement. We therefore devised a recreation of the PDD measurements wherein we collected individual PDD values with the chamber located statically at each point within the same tank (i.e., Scanditronix/Wellhöfer system with internal RFA‐300 electrometer controller). This approach eliminated scanning motor operation and, theoretically, the potential for contribution of motor noise to the measurement anomaly. During this experiment we also repeated measurements using two different, independent electrometers (Keithley Model MK602 serial #280033 (Keithley, Cleveland, OH) and Standard Imaging Model Max4000 serial #E070804) and collection cables to measure current, in an attempt to determine the role of the electrometer in the observed phenomenon (Setup E from Table 1).

**Figure 6:**
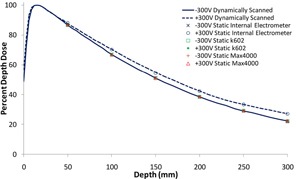
Comparison between percent depth doses (PDDs) from a 6 MV flattened beam obtained dynamically and statically. All data were obtained using an A16 microchamber operated at biases of positive AND negative 300V in the Scanditronix/Wellhöfer water tank. ‘Static, Internal Electrometer’ data used the Scanditronix/Wellhöfer's internal RFA‐300 electrometer. The dynamic data was obtained using the water tank to ‘scan’ the PDD. Static measurements were obtained by first driving the ion chamber to known, statically located depths and then using the RFA‐300 internal electrometer to obtain the raw current reading. Additionally, external electrometers (MK602 and a MAX 4000) were also used to provide bias to the chamber and obtain current readings with the chamber being static. The current measurements were then used to obtain the percent depth dose for each depth. The reader should observe that the dynamic scan acquired with negative bias and internal electrometer (solid line), and all static scans acquired with external electrometer overlay each other with excellent agreement, thus indicating that no PDD measurement anomaly exists when either a negative bias is used or when a scanner‐independent, external electrometer is used. The figure shows that only when the scanning system's internal electrometer is used, in positive bias mode, does the anomaly manifest (dashed ‘scanned’ line and circle symbols for statically acquired data). The fact that the anomaly manifests for both static and dynamic modes indicates that operation of the scanning motors does not contribute to the phenomenon.

A summary of results for these experiments is presented here in Fig. 6 and shows that, when the PDD is sampled statically using readings from the water tank's internal electrometer (RFA‐300), the PDD curve does demonstrate the discrepancy when the bias is changed. We also note that, in a separate experiment (data not shown), this same behavior was also confirmed when using the PTW MP3‐M scanning system with internal electrometer, when negative bias was used. In contrast, Fig. 6 results using the external, independent MK602 and Max4000 electrometers show that both positive and negative bias static PDD values overlay extremely well with the negative bias scanned curves for the 6 MV beam, with these results also holding true for the 10 MV flattened, 6 MV FFF, and 10 MV FFF beams; in other words, the measurement difference was no longer observable when an external electrometer was used.

It is clear from Fig. 6 that the ‘outlier’ curve is only produced when the positive 300V bias is used, in combination with the scanning system's controller and electrometer, which we postulate to be a result of signal interference generated in the controller box being collected or registered by the measurement system. We further postulate that it is reasonable to see the phenomena manifested to greatest degree in smaller chambers, where the collected ‘primary’ signal is small enough that the relative contribution from noise/interference can become significant. The increase in measurement difference as a function of depth is also reasonable, given that the primary signal only decreases further as the chamber is scanned to depth.

Table 2 presents current values in pA for a sampling of statically located ion chambers used in this work, at depths of 1.5 cm ($d_{\text{max}}$) and 25 cm, when the beam was operated at a relatively high dose rate of 640 MU/min in a 6 MV flattened beam. The table demonstrates both the significant reduction in signal magnitude at 25 cm depth when changing from, for instance, a 0.3 cc volume chamber (∼84 pA) to a so‐called ‘micro’ chamber volume of 0.007 cc (∼7 pA), along with presenting the nontrivial falloff in signal in going from $d_{\text{max}}$ to 25 cm depth. Assuming that the postulated contribution from the controller/electrometer system‐induced interference is relatively constant as a function of chamber location/depth, the relative contribution from noise will obviously increase significantly for smaller chambers, and as a function of increasing depth.

**Table 2:** Current measured using a statically located chamber with a CNMC MK602 electrometer when the 6X beam was operated at a relatively high dose rate of 640 MU/min

*Ion Chamber*	*Ion Chamber Volume (cc)*	*Bias Set (V)*	*Current at d_max_ (pA)*	*Current at 25 cm (pA)*
PTW 31013	0.3	−300 300	−303.4 301.2	−84.9 84.3
Exradin A1SL	0.053	−300 300	−144.3 144.1	−40.4 40.3
Wellhöffer IC04	0.04	−300 300	−89.8 90.3	−25.0 25.1
Exradin A16 #1	0.007	−300 300	−20.7 20.6	−5.8 5.8
Exradin A16 #2	0.007	−300 300	−27.3 27.4	−7.7 7.7
Exradin A16 #3	0.007	−300 300	−21.3 21.2	−6.0 6.0

It may seem reasonable to attribute our observed discrepancy between the two PDD curves acquired at ± 300V to a polarity effect.^19^, ^20^, ^21^ A polarity effect is characterized by different readout signals from ion chambers when opposite voltages were applied, a phenomenon investigated in the mid‐1980s by Gerbi and Khan and a difference up to 30% was shown in the buildup region of high energy photon beams for plane‐parallel ion chambers.^(2)^ Later, the polarity effect was studied by Ramsey et al.^(22)^ for electron beams, but its magnitude was less than 1% at $d_{\text{max}}$ for plane‐parallel chambers and no polarity effect was observed for cylindrical chambers. Another study found strong dependency of the polarity effect on the sensitive volume of an ion chamber for kilovoltage beams.^(23)^ However, we believe that our observed discrepancy is not related to the polarity effect, as defined in the literature, because the discrepancy disappears when measured using an external electrometer system.

Another possible effect that could play a role in the effect is the stem effect.^(24)^ Given the extremely small size of the active volume of the A16 chamber, a large portion of the stem is necessarily included in the $10\times 10\;{\text{cm}}^{2}$ field used in gathering the data. However, we do not believe that the stem effect is responsible for the discrepancy seen here. This is because the exact same amount of stem was exposed when the experiment was performed to check for the effect when using the internal scanning system electrometer and the independent external electrometer. The effect was observed in one case but not in the other, thus indicating that the stem effect cannot be the culprit.

The material used in the chamber construction was also explored as a potential source of the anomalous effect observed here. The Exradin A16, Wellhöffer IC04, Exradin A1SL, Exradin A28, and IBA CC13 chambers all use C552 Shonka plastic for their shell, collector, and guard. The PTW 31010, PTW 31013, and PTW N30013 chambers have a PMMA and graphite shell with an aluminum electrode. While both chambers that exhibit the effect (Exradin A16 and Wellhöffer IC04) use Shonka plastic, the larger CC13 and A28 chambers are also made of the same material and do not exhibit the effect. Therefore, it does not appear that chamber construction is involved with this effect.

The behavior of very small volume chambers has been investigated by several authors.^17^, ^24^, ^25^ McEwen^(25)^ previously recommended against using these chambers for the purposes of absolute dosimetry, but the anomaly reported here shows that, even in relative dosimetry, there is a potential to introduce systematic errors in the beam model created from scans obtained using these microchambers. While there exists an undeniable potential benefit to using very small active volume chambers to better characterize the penumbra characteristics of any beam, the use of these relatively new chambers must also carefully consider the scanning environment and the potential for introduction of measurement anomalies, such as were observed here. We note that the anomalous PDD is obtained when a +300V bias was used in the Scanditronic/Wellhöfer tank and the Standard Imaging DoseView 3D tank, but when using the −300V bias in the PTW MP3‐M tank. One possible explanation is that the bias convention used in the PTW tank differs from that used in the other two tanks. However, when a voltmeter was used to determine what voltage was being applied to each terminal of the triax connector, when the same biases were selected in the software for the Scanditronix/Wellhöfer tank and the PTW tank, the same potential difference was found to exist within the connector. This suggests that the source of the error is not due to opposite bias conventions between manufacturers. Since the bias required to obtain presumed “true” scans varies with scanning system, we cannot make specific recommendations on which bias should be used when scanning a beam.

We do, however, recommend that whenever such very small ion chambers are to be used, the user should obtain a set of initial PDD scans for a representative field size using a larger chamber for comparison with both positive and negative bias PDD curves obtained with the small chamber. If the PDD anomaly reported here exists for the user's particular scanning system/internal electrometer/ion chamber combination, it will be apparent as nontrivial disagreement between the large ion chamber PDD curve (ground truth) and one of the positive/negative bias scans from the small chamber. At that point the user can know which electrometer bias to avoid, if any, for their particular equipment combination. This test should be done for a medium field size in order to make sure that the entire active volume of the larger chamber is being irradiated and the scan can be compared to the ones obtained using the smaller chamber.

While we cannot give a thorough explanation as to why this behavior is observed, the general normal shape of the anomalous PDD shows that this phenomenon is very insidious in its presentation, but has the potential of introducing a systematic mischaracterization of the beam on the order of 3%–4% at depth. Given that we have shown this effect to occur in multiple scanning systems, we believe it is extremely important that anyone considering using any microchamber (especially one of the chambers that have shown the abnormality in our investigation) follow, at the very least, the recommended approach of determining whether changing the bias on the water tank leads to vastly differing scans.

## V. CONCLUSIONS

The purpose of this paper is to characterize and present our observation of anomalous PDD scans obtained using specific water tank scanners/microchamber combinations. The anomaly observed here has the potential for introducing nontrivial systematic errors into the treatment planning process, but can be avoided by acquiring preliminary ‘test’ scans recommended herein. Awareness of the anomaly is important for anyone considering use of such chambers for measuring percent depth‐dose curves for purposes of commissioning a treatment planning system.

## ACKNOWLEDGMENTS

The authors wish express their gratitude to Dr. Keith Welsh for loaning us ion chamber equipment for these experiments. We also thank Rebekah Hansen and Alex Richetta of Standard Imaging for their assistance in obtaining data in their DoseView 3D tank.

## References

[1] Sibata CH , Mota HC , Beddar AS , Higgins PD , Shin KH . Influence of detector size in photon beam profile measurements. Phys Med Biol. 1991;36(5):621–31. 2068227 10.1088/0031-9155/36/5/005

[2] Laub WU and Wong T . The volume effect of detectors in the dosimetry of small fields used in IMRT. Med Phys. 2003;30(3):341–47. 12674234 10.1118/1.1544678

[3] Griessbach I , Lapp M , Bohsung J , Gademann G , Harder D . Dosimetric characteristics of a new unshielded silicon diode and its application in clinical photon and electron beams. Med Phys. 2005;32(12):3750–54. 16475774 10.1118/1.2124547

[4] McKerracher C and Thwaites DI . Assessment of new small‐field detectors against standard‐field detectors for practical stereotactic beam data acquisition. Phys Med Biol. 1999;44(9):2143–60. 10495110 10.1088/0031-9155/44/9/303

[5] Das IJ , Cheng CW , Watts RJ , et al. Accelerator beam data commissioning equipment and procedures: report of the TG‐106 of the Therapy Physics Committee of the AAPM. Med Phys. 2008;35(9):4186–215. 18841871 10.1118/1.2969070

[6] Rikner G and Grusell E . General specifications for silicon semiconductors for use in radiation dosimetry. Phys Med Biol. 1987;32(9):1109–17. 3671497 10.1088/0031-9155/32/9/004

[7] Georg D , De Ost B , Hoornaert MT , et al. Build‐up modification of commercial diodes for entrance dose measurements in ‘higher energy’ photon beams. Radiother Oncol. 1999;51(3):249–56. 10435820 10.1016/s0167-8140(99)00058-4

[8] Saini AS and Zhu TC . Energy dependence of commercially available diode detectors for in‐vivo dosimetry. Med Phys. 2007;34(5):1704–11. 17555252 10.1118/1.2719365

[9] Saini AS and Zhu TC . Temperature dependence of commercially available diode detectors. Med Phys. 2002;29(4):622–30. 11991134 10.1118/1.1461842

[10] Saini AS and Zhu TC . Dose rate and SDD dependence of commercially available diode detectors. Med Phys. 2004;31(4):914–24. 15125010 10.1118/1.1650563

[11] Hoban PW , Heydarian M , Beckham WA , Beddoe AH . Dose rate dependence of a PTW diamond detector in the dosimetry of a 6 MV photon beam. Phys Med Biol. 1994;39(8):1219–29. 15551563 10.1088/0031-9155/39/8/003

[12] Laub WU , Kaulich TW , Nusslin F . Energy and dose rate dependence of a diamond detector in the dosimetry of 4‐25 MV photon beams. Med Phys. 1997;24(4):535–36. 9127304 10.1118/1.597902

[13] Vatnitsky S and Jarvinen H . Application of a natural diamond detector for the measurement of relative dose distributions in radiotherapy. Phys Med Biol. 1993;38(1):173–84. 8381236 10.1088/0031-9155/38/1/013

[14] Guillot M , Gingras L , Archambault L , Beddar S , Beaulieu L . Performance assessment of a 2D array of plastic scintillation detectors for IMRT quality assurance. Phys Med Biol. 2013;58(13):4439–54. 23756497 10.1088/0031-9155/58/13/4439

[15] Morin J , Beliveau‐Nadeau D , Chung E , et al. A comparative study of small field total scatter factors and dose profiles using plastic scintillation detectors and other stereotactic dosimeters: the case of the CyberKnife. Med Phys. 2013;40(1):011719. 23298089 10.1118/1.4772190

[16] Gagnon J‐C , Theriault D , Guillot M , et al. Dosimetric performance and array assessment of plastic scintillation detectors for stereotactic radiosurgery quality assurance. Med Phys. 2012;39(1):429–36. 22225313 10.1118/1.3666765

[17] Stasi M , Baiotto B , Barboni G , Scielzo G . The behavior of several microionization chambers in small intensity modulated radiotherapy fields. Med Phys. 2004;31(10):2792–95. 15543786 10.1118/1.1788911

[18] Martens C , De Wagter C , De Neve W . The value of the PinPoint ion chamber for characterization of small field segments used in intensity‐modulated radiotherapy. Phys Med Biol. 2000;45(9):2519–30. 11008953 10.1088/0031-9155/45/9/306

[19] Crespi A , Zavatti M , Tremolada V , De Ponti E , Montanari G , Paruccini N . The polarity effect for different PTW ionization chambers under various irradiation conditions. Phys Med. 1997;13(1):25–30.

[20] Fiorino C , Mangili P , Cattaneo GM , Calandrino R . Polarity effects of ionization chambers used in TBI dosimetry due to cable irradiation. Med Dosim. 2000;25(3):121–26. 11025257 10.1016/s0958-3947(00)00037-6

[21] Shimono T , Koshida K , Nambu H , et al. Polarity effect in commercial ionization chambers used in photon beams with small fields. Radiol Phys Technol. 2009;2(1):97–103. 20821135 10.1007/s12194-008-0050-1

[22] Ramsey CR , Spencer KM , Oliver AL . Ionization chamber, electrometer, linear accelerator, field size, and energy dependence of the polarity effect in electron dosimetry. Med Phys. 1999;26(2):214–19. 10076977 10.1118/1.598507

[23] Das IJ and Akber SF . Ion recombination and polarity effect of ionization chambers in kilovoltage x‐ray exposure measurements. Med Phys. 1998;25(9):1751–57. 9775383 10.1118/1.598360

[24] Ma CM and Nahum AE . Monte Carlo calculated stem effect correction for NE2561 and NE2571 chambers in medium‐energy x‐ray beams. Phys Med Biol. 1995;40(1):63–72. 7708844 10.1088/0031-9155/40/1/006

[25] McEwen MR . Measurement of ionization chamber absorbed dose k(Q) factors in megavoltage photon beams. Med Phys. 2010;37(5):2179–93. 20527552 10.1118/1.3375895

